# Efficacy and safety of intensive vs. standard blood pressure control: a systematic review and meta-analysis

**DOI:** 10.3389/fcvm.2026.1929546

**Published:** 2026-09-23

**Authors:** Huarong Yang, Yang Li, Guiyuan Xiang, Shuchen Pei, Man Wang, Lihong Xu, Bingbing Zhu, Yao Liu, Xue Yang

**Affiliations:** 1 Department of Pharmacy, Daping Hospital, Army Medical University, Chongqing, China; 2 School of Chemistry and Chemical Engineering, Chongqing University of Science and Technology, Chongqing, China

**Keywords:** hypertension, intensive blood pressure control, meta-analysis, randomized controlled trial, standard blood pressure control

## Abstract

**Objective:**

The study aimed to compare the efficacy and safety of intensive systolic blood pressure (SBP) control (target SBP < 120 mmHg) vs. standard blood pressure control (target SBP < 140 mmHg) in reducing cardiovascular risk.

**Methods:**

We conducted a systematic search of the Cochrane Library, Embase, and PubMed databases, covering the period from the inception of each database to February 11, 2025, to identify randomized controlled trials comparing these two blood pressure control targets. Efficacy outcomes included major adverse cardiovascular events, heart failure, stroke, myocardial infarction, cardiovascular mortality, and all-cause mortality. Safety outcomes included hypotension, syncope, falls, and renal dysfunction.

**Results:**

Five randomized controlled trials involving 39,433 patients were included. Compared to standard blood pressure control, intensive blood pressure control was associated with a significant reduction in the risk of major adverse cardiovascular events (RR, 0.80; 95% CI, 0.75–0.86; *P* < 0.001), heart failure (RR, 0.74; 95% CI, 0.62–0.90; *P* = 0.002), stroke (RR, 0.81; 95% CI, 0.73–0.90; *P* < 0.001), cardiovascular mortality (RR, 0.73; 95% CI, 0.56–0.94; *P* = 0.01), and all-cause mortality (RR, 0.86; 95% CI, 0.75–0.99; *P* = 0.03). Intensive blood pressure control was associated with a borderline statistically significant reduction in the risk of myocardial infarction (RR, 0.87, 95% CI 0.76–1.00; *P* = 0.04). Intensive blood pressure control increased the risks of hypotension (RR, 3.63; 95% CI, 1.41–9.35; *P* = 0.008) and syncope (RR, 1.50; 95% CI, 1.22–1.85; *P* < 0.001).

**Conclusion:**

Based on currently available evidence, intensive blood pressure control may reduce the risk of cardiovascular events and mortality compared to standard blood pressure control, but it may increase the risk of hypotension and syncope.

## Introduction

The World Health Organization estimates that between 1990 and 2019, the number of people aged 30 to 79 with hypertension worldwide doubled, rising from 650 million in 1990 to 1.28 billion in 2019 (1). From 1991 to 2015, the age-adjusted prevalence of hypertension among Chinese residents increased from 15.3% to 25.6% (2). In the United States, the age-adjusted prevalence of hypertension among adults aged 20 and older was estimated at 46.7% from 2017 to 2020 (3). Elevated systolic blood pressure is not only the most prevalent and modifiable risk factor for cardiovascular diseases (4, 5), but also the leading global risk factor for death and disability, affecting over one billion people and causing an estimated 10.4 million deaths annually (6). Antihypertensive treatment can significantly reduce the risk of major adverse cardiovascular events, coronary heart disease, stroke, heart failure, and all-cause mortality (7–10). However, the proportion of hypertensive patients achieving their blood pressure target remains unacceptably low, which is a persistent public health concern (11). This underscores a critical question: what is the optimal blood pressure target for hypertensive patients? The traditional antihypertensive target is to control the systolic blood pressure below 140 mmHg. Over the past decade, growing evidence has strongly supported lowering systolic blood pressure to below 130 mmHg (12–15). Meta-analyses have further demonstrated that intensive blood pressure control reduces the risk of cardiovascular events (16–18). Based on this evidence, recent major guidelines have shifted towards recommending more stringent blood pressure targets and lower treatment thresholds compared to their predecessors. The 2024 ESC guidelines recommend a target systolic blood pressure range of 120–129 mmHg for most treated adults (19). The 2025 AHA/ACC guidelines recommend a unified target of <130/80 mmHg for all adults with hypertension (20). It is well known that traditionally, systolic blood pressure below 120 mmHg and diastolic blood pressure below 80 mmHg have been defined as normal blood pressure. The ACCORD study aimed to reduce systolic blood pressure to below 120mmHg, but no benefits were observed in the composite outcome of fatal and non-fatal major cardiovascular events (21). The results of the subsequent SPRINT study indicated that setting a systolic blood pressure target below 120 mmHg could reduce the incidence of major fatal and non-fatal cardiovascular events and all-cause mortality (22). Therefore, a key question remains: Would further lowering systolic blood pressure to the normal range (target SBP <120 mmHg) provide additional benefit? To address this question, we conducted this meta-analysis to evaluate the efficacy and safety of intensive blood pressure control (target SBP < 120 mmHg) compared with standard blood pressure control (target SBP < 140 mmHg). It is important to clarify that the threshold of SBP <140 mmHg represents the predefined comparator target adopted by the majority of the included landmark trials, rather than a reflection of the contemporary guideline-recommended standard of care.

## Methods

### Data sources and searches

This systematic review and meta-analysis was conducted in accordance with the Preferred Reporting Items for Systematic Reviews and Meta-Analyses (PRISMA) guidelines (23). A comprehensive literature search was performed in the Cochrane Library, PubMed, and Embase databases from their inception through February 11, 2025. We included randomized controlled trials that compared the efficacy and safety of intensive vs. standard blood pressure control strategies on cardiovascular outcomes. The search strategy used “Hypertension” as a Medical Subject Heading term, along with keywords including “intensive blood pressure control”, “antihypertensive”, and “cardiovascular events”. The search was limited to randomized controlled trials published in English. During the literature search process, grey literature, clinical trial registries, unpublished data and ongoing trials have not been retrieved. The detailed search strategy for each database is provided in the Supplementary Tables S1–S3*.* Two independent reviewers conducted the literature search and selection. Additionally, the reference lists of all included studies and relevant review articles were manually screened to identify potentially eligible publications.

### Study selection

Two reviewers independently screened the titles and abstracts of all retrieved records. The studies deemed potentially relevant were then evaluated in full text by the same two reviewers. Any disagreements regarding eligibility were resolved through discussion or, when necessary, a third reviewer was consulted for a final decision. Studies were included if they met the following criteria: (1) randomized controlled trials comparing intensive blood pressure control with standard blood pressure control (In our meta-analysis SBP <140 mmHg is a pre-defined control target used in the original included trials rather than a reflection of current guideline-recommended standard practice). Studies were excluded for any of the following reasons: (1) the primary objective of the study was not to evaluate the difference between intensive and standard blood pressure control; (2) the study population included patients under 18 years of age; (3) the study design was not randomized controlled trial (e.g., case reports, reviews, cohort studies); (4) the study did not report any of the predefined outcomes of interest for this meta-analysis; (5) the study for which raw data or data sufficient for analysis were not available (e.g., only published in abstract form); (6) the intervention or population fundamentally differed from the focus of this review (e.g., acute hypertensive management, secondary hypertension only).

### Outcome variables

The primary efficacy outcome was a composite of major adverse cardiovascular events, including fatal or non-fatal heart failure, myocardial infarction, stroke, and cardiovascular mortality. To minimize heterogeneity arising from variable composite endpoint definitions, we did not use the trial-reported composite endpoints directly. Instead, we extracted individual component-level data (event counts for heart failure, myocardial infarction, stroke, and cardiovascular mortality) from each trial's primary publication or Supplementary materials. A harmonized four-component major adverse cardiovascular events was then reconstructed for each trial prior to pooling.

Secondary efficacy outcomes included all-cause mortality and the individual components of the primary composite outcome (heart failure, myocardial infarction, stroke, and cardiovascular mortality). The primary safety outcome was hypotension, while the secondary safety outcomes included syncope, falls, and renal dysfunction.

### Data extraction and quality assessment

Data extraction was performed independently by two reviewers, any disagreements were resolved by consensus or by consulting a third reviewer. The following data were extracted: Study characteristics: follow-up period, sample size. Participant baseline information: mean age, baseline blood pressure, prevalence of diabetes, and history of stroke. Efficacy outcomes: number of events for the primary composite outcome and its individual components, including heart failure, myocardial infarction, stroke, cardiovascular mortality, and all-cause mortality. Safety outcomes: number of events for hypotension, syncope, falls, and renal dysfunction. The risk of bias in the included randomized controlled trials was evaluated using the Cochrane Risk of Bias 2.0 (RoB 2) tool (24). The certainty of evidence for each outcome was assessed using the GRADE framework (25).

### Data synthesis and analysis

All statistical analyses were performed using Review Manager (RevMan) version 5.4 (The Cochrane Collaboration, Nordic Cochrane Centre, Denmark). The pre-specified random-effects model was adopted to conduct a meta-analysis of all pooled effect sizes. For each outcome, we included only those trials that reported the relevant outcome data. A complete summary of outcome-specific trial inclusions and exclusions, with explicit reasons for each exclusion, is provided in Supplementary Table S4. For all dichotomous outcomes, the treatment effects are presented as risk ratios (RR) with 95% confidence intervals (CI). Sensitivity analysis was conducted by sequentially excluding each individual study to evaluate the robustness of the pooled results. To enhance the clinical interpretability of our findings, we calculated absolute risk differences (ARDs) and numbers needed to treat (NNTs) or harm (NNHs) for all outcomes. To assess the robustness of the pooled estimates and to control for random error due to sparse data and repeated significance testing, we performed Trial Sequential Analysis (TSA) (26).

## Results

### Study selection and characteristics

The study selection process is detailed in the PRISMA flow diagram (Figure 1). Initial searches across three databases yielded a total of 5,011 records. After excluding 309 duplicates, 4,661 records were excluded during title and abstract screening. Following full-text evaluation, 5 randomized controlled trials ultimately met the inclusion criteria and were included in the meta-analysis (21, 22, 27–29). These 5 randomized controlled trials enrolled a total of 39,433 participants, with individual study sample sizes ranging from 1,263 to 12,821. Their baseline characteristics are presented in Table 1. The study populations varied: two trials specifically investigated intensive blood pressure control in patients with type 2 diabetes (21, 29), while one trial explicitly excluded participants with diabetes (22); One trial focused on patients with a prior stroke (27), and another included high cardiovascular-risk patients, irrespective of diabetes or stroke history (28).

**Figure 1:**
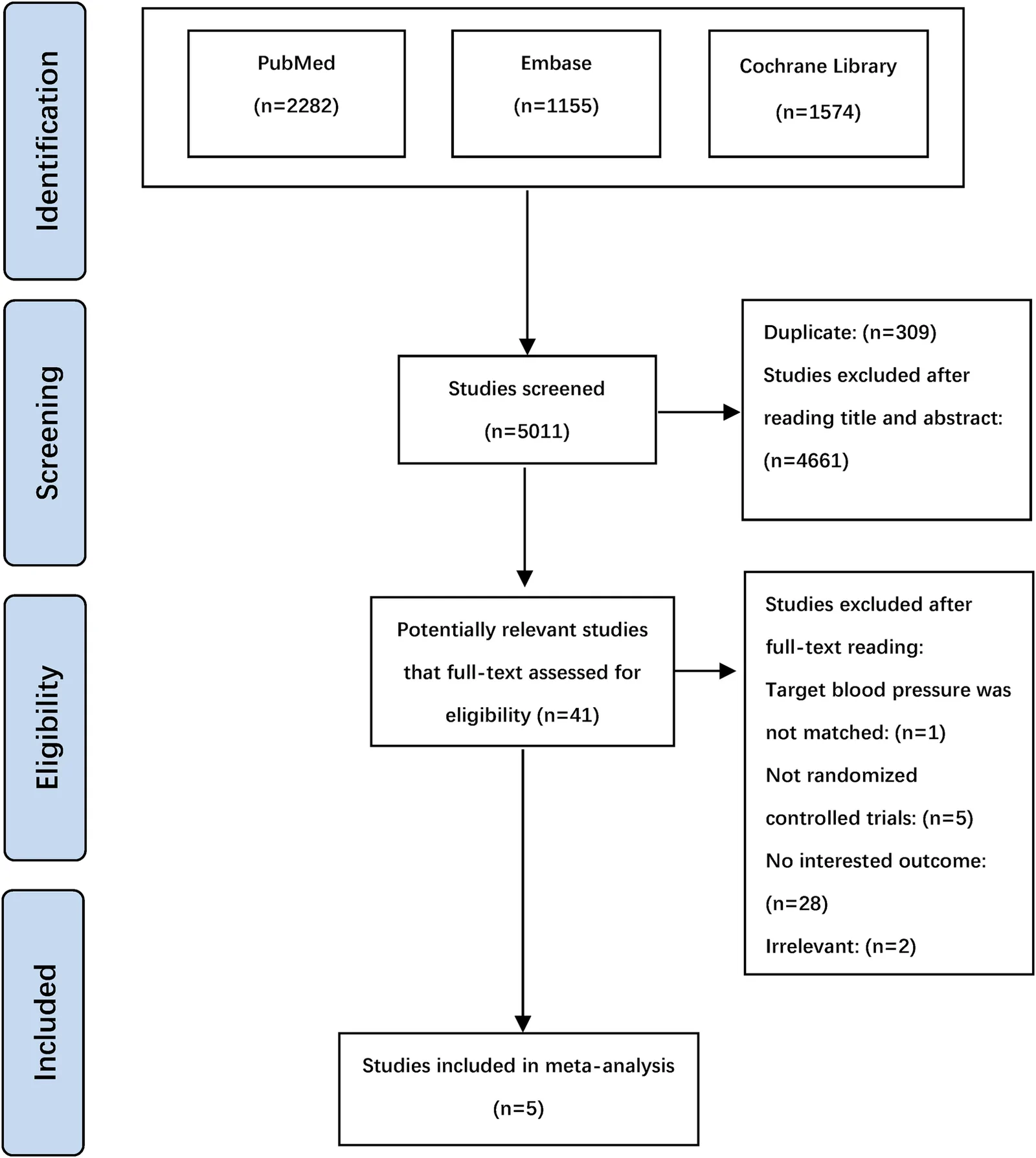
Flowchart of study selection.

**Table 1:** Baseline characteristics of included studies.

Study, NCT	Follow up （year）	Participants(n)		Age(year)		Baseline blood pressure（mmHg）		Diabetes（%）		Stroke(%)		Blood pressure after treatment（mmHg）	
		intensive	standard	intensive	standard	intensive	standard	intensive	standard	intensive	standard	intensive	standard
ACCORD, NCT00000620	4.7	2362	2371	62.2 ± 6.8	62.2 ± 6.9	139.0/75.9	139.4/76.0	100.0	100.0	-	-	119.3/64.4	133.5/70.5
SPRINT, NCT01206062	3.26	4678	4683	67.9 ± 9.4	67.9 ± 9.5	139.7/78.2	139.7/78.0	-	-	-	-	121.4/68.7	136.2/76.3
RESPECT, NCT01198496	3.9	633	630	67.2 ± 8.8	67.3 ± 8.8	145.1/83.6	145.7/83.7	22.4	24.4	100.0	100.0	123.7/72.8	132.0/77.5
ESPRIT, NCT04030234	3.4	5624	5631	64.6 ± 7.1	64.6 ± 7.2	146.8/82.8	147.0/82.9	38.8	38.7	27.0	26.7	119.1/-	134.8/-
BPROAD, NCT03808311	4.2	6414	6407	63.7 ± 7.4	63.9 ± 7.5	140.0/76.3	140.4/76.3	100.0	100.0	13	12.1	121.6/-	133.2/-

‘-/-’ denotes data not reported in the original studies.

The methodological quality of the included randomized controlled trials was assessed using the Cochrane RoB 2. All trials were open-label, and patients were aware of their treatment assignment; consequently, the domain of deviations from intended interventions was rated as Some concerns. Thus, the overall risk of bias for the primary efficacy outcomes was judged as Some concerns. The detailed domain-level assessments and overall judgments are presented in Supplementary Figure S1. The GRADE certainty ratings for all outcomes are summarized in Supplementary Table S5. For the primary major adverse cardiovascular events outcome and most secondary efficacy outcomes, the certainty of evidence was rated as High. However, the certainty was downgraded to Moderate for cardiovascular mortality (I^2^ = 54%) and for hypotension (I^2^ = 66%), due to considerable statistical heterogeneity across studies.

### Outcomes of efficacy

A total of 4 studies (involving 38,170 patients) provided data for the analysis of the primary efficacy outcome. Intensive blood pressure control significantly reduced the risk of major adverse cardiovascular events by 20% (RR, 0.80; 95% CI, 0.75–0.86; *P* < 0.001; I^2^ = 0%) (Figure 2). The corresponding numbers needed to treat to prevent one major adverse cardiovascular events was 53 over 3.8 years, based on an absolute risk reduction of 19 fewer events per 1,000 patients (Supplementary Table S6). As shown in Figure 3A, intensive blood pressure control was associated with a significantly lower risk of heart failure (RR, 0.74; 95%CI, 0.62–0.90; *P* = 0.002; I^2^ = 17%). In the subgroup analysis, intensive blood pressure control did not significantly reduce the risk of fatal heart failure compared with standard blood pressure control (RR,0.92; 95%CI, 0.52–1.63; *P* = 0.78; I^2^ = 0%) (Figure 3B). The observed benefit was primarily driven by a reduction in the risk of non-fatal heart failure (RR, 0.72; 95%CI, 0.55–0.95; *P* = 0.02; I^2^ = 40%) (Figure 3C). As shown in Figure 4A, intensive blood pressure control significantly reduced the risk of stroke (RR,0.81; 95%CI, 0.73–0.90; *P* < 0.001; I^2^ = 0%). Subgroup analysis based on stroke severity revealed a non-significant risk reduction for fatal stroke (RR, 0.70; 95%CI, 0.49–1.01; *P* = 0.06; I^2^ = 0%) (Figure 4B), whereas a statistically significant protective effect was observed in the non-fatal stroke subgroup (RR, 0.83; 95%CI, 0.74–0.92; *P* < 0.001; I^2^ = 0%) (Figure 4C). As shown in Figure 5A, intensive blood pressure control exerted a borderline statistically significant reducing effect on the risk of myocardial infarction (RR, 0.87; 95%CI, 0.76–1.00; *P* = 0.04; I^2^ = 0%). Due to the availability of data from only a single study, meaningful subgroup analysis of fatal and non-fatal events was not feasible. As shown in Figure 6, intensive blood pressure control was associated with a significant reduction in both all-cause mortality (RR, 0.86; 95%CI, 0.75–0.99; *P* = 0.03; I^2^ = 40%) and cardiovascular mortality (RR, 0.73; 95% CI, 0.56–0.94; *P* = 0.01; I^2^ = 54%).

**Figure 2:**
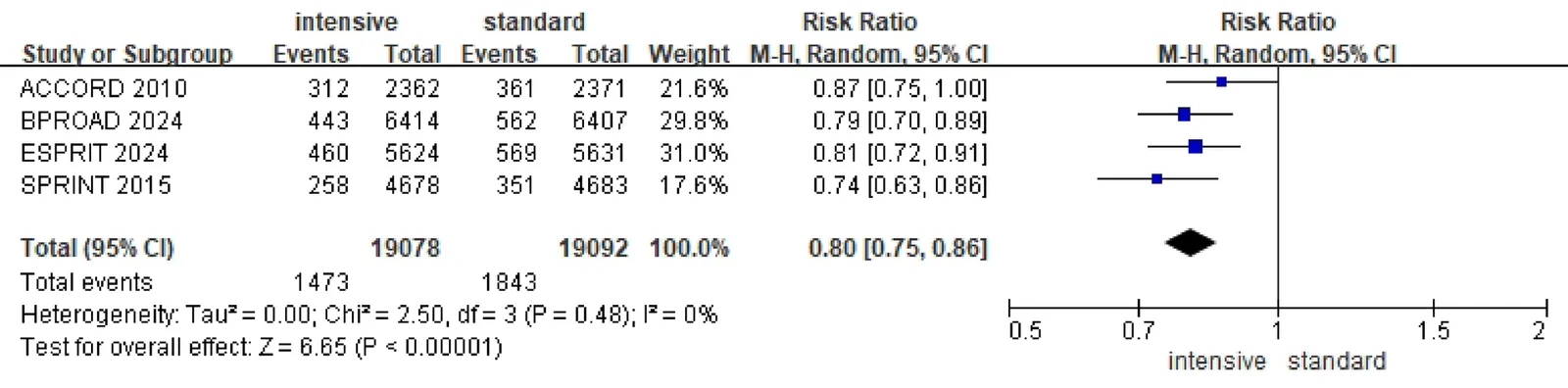
Effect of intensive blood pressure control on major adverse cardiovascular events.

**Figure 3:**
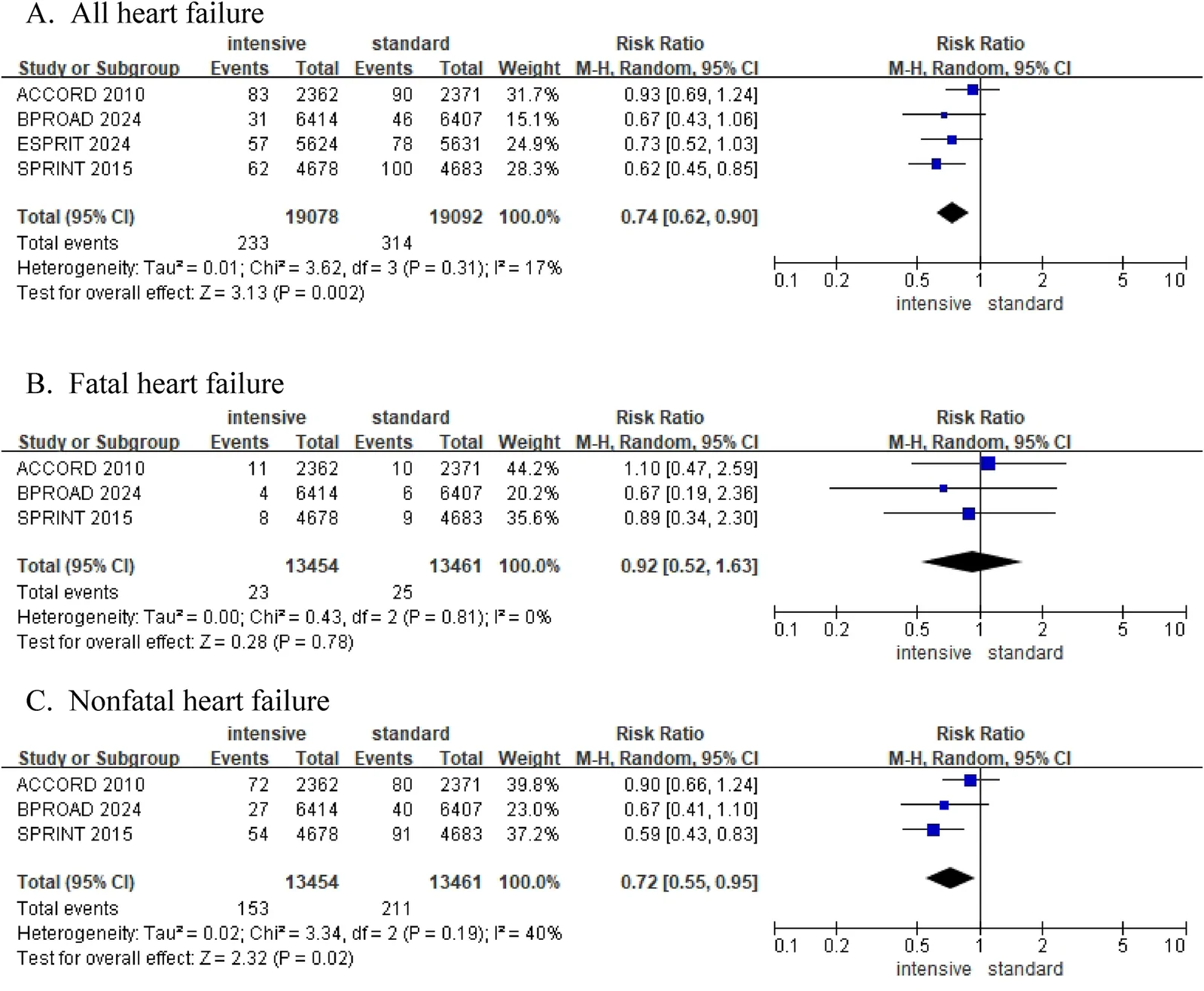
Effect of intensive blood pressure control on heart failure. **A**. All heart failure. **B**. Fatal heart failure. **C**. Nonfatal heart failure.

**Figure 4:**
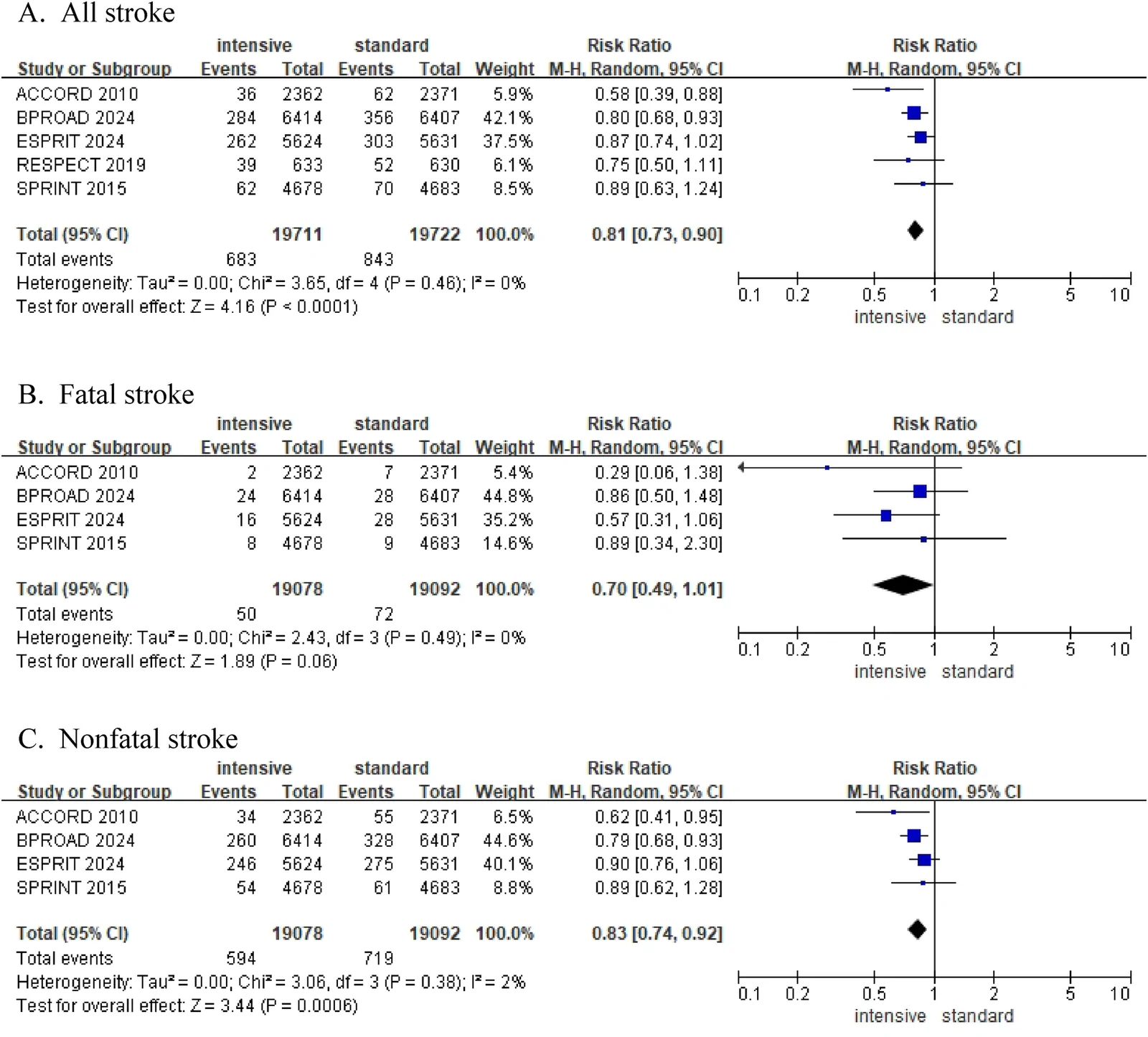
Effect of intensive blood pressure control on stroke. **A**. All stroke. **B**. Fatal stroke. **C**. Nonfatal stroke.

**Figure 5:**
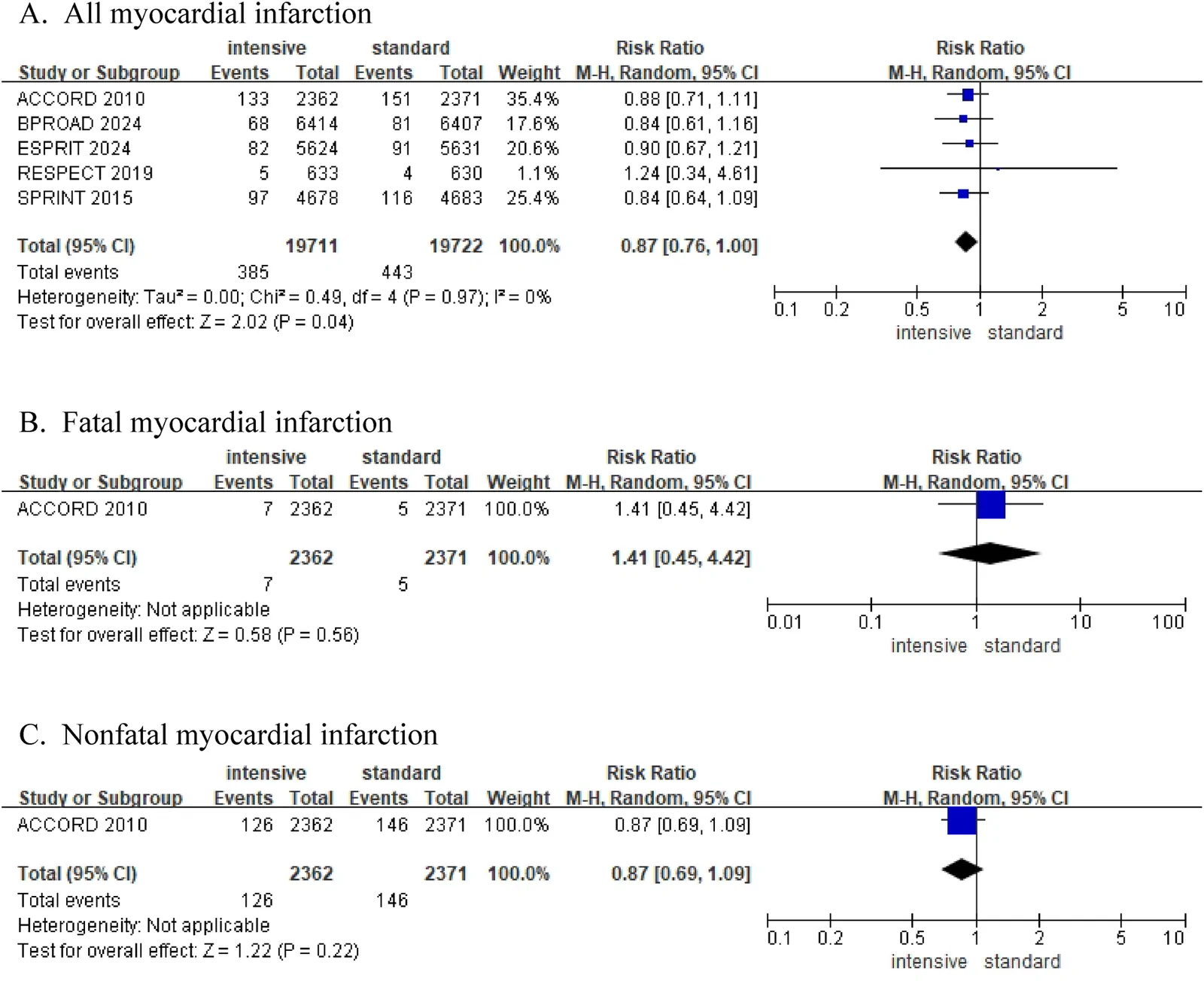
Effect of intensive blood pressure control on myocardial infarction. **A**. All myocardial infarction. **B**. Fatal myocardial infarction. **C**. Nonfatal myocardial infarction.

**Figure 6:**
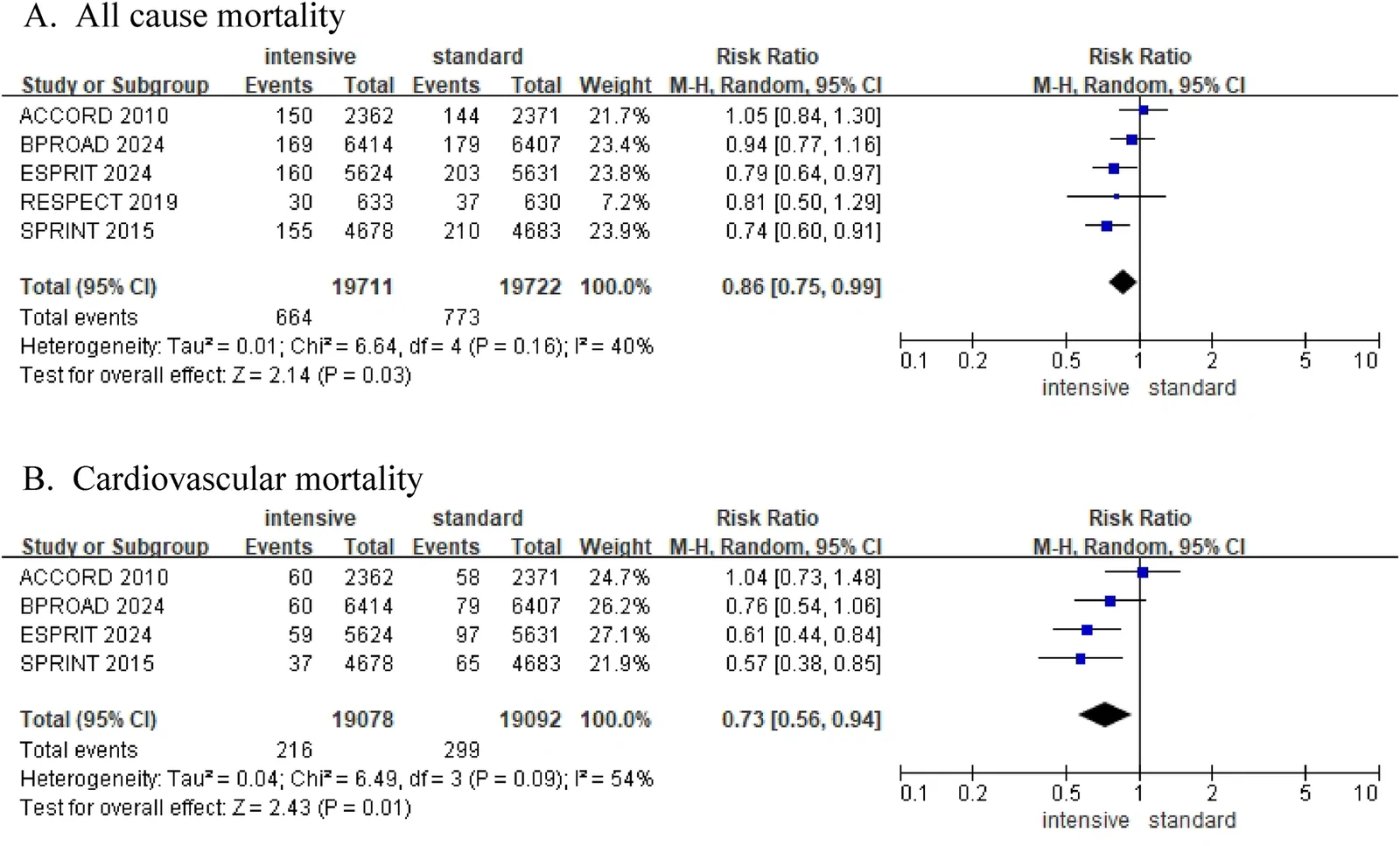
Effect of intensive blood pressure control on mortality. **A**. All cause mortality. **B**. Cardiovascular mortality.

### Outcomes of safety

As shown in Figure 7, intensive blood pressure control was associated with a significantly increased risk of hypotension (RR, 3.63; 95%CI, 1.41–9.35; *P* = 0.008; I^2^ = 66%) and syncope (RR, 1.50; 95%CI, 1.22–1.85; *P* < 0.001; I^2^ = 0%), although it did not significantly increase the risk of falls (RR, 1.03; 95%CI, 0.90–1.17; *P* = 0.65; I^2^ = 0%) and renal dysfunction (RR, 1.41; 95%CI, 0.98–2.02; *P* = 0.07; I^2^ = 54%).

**Figure 7:**
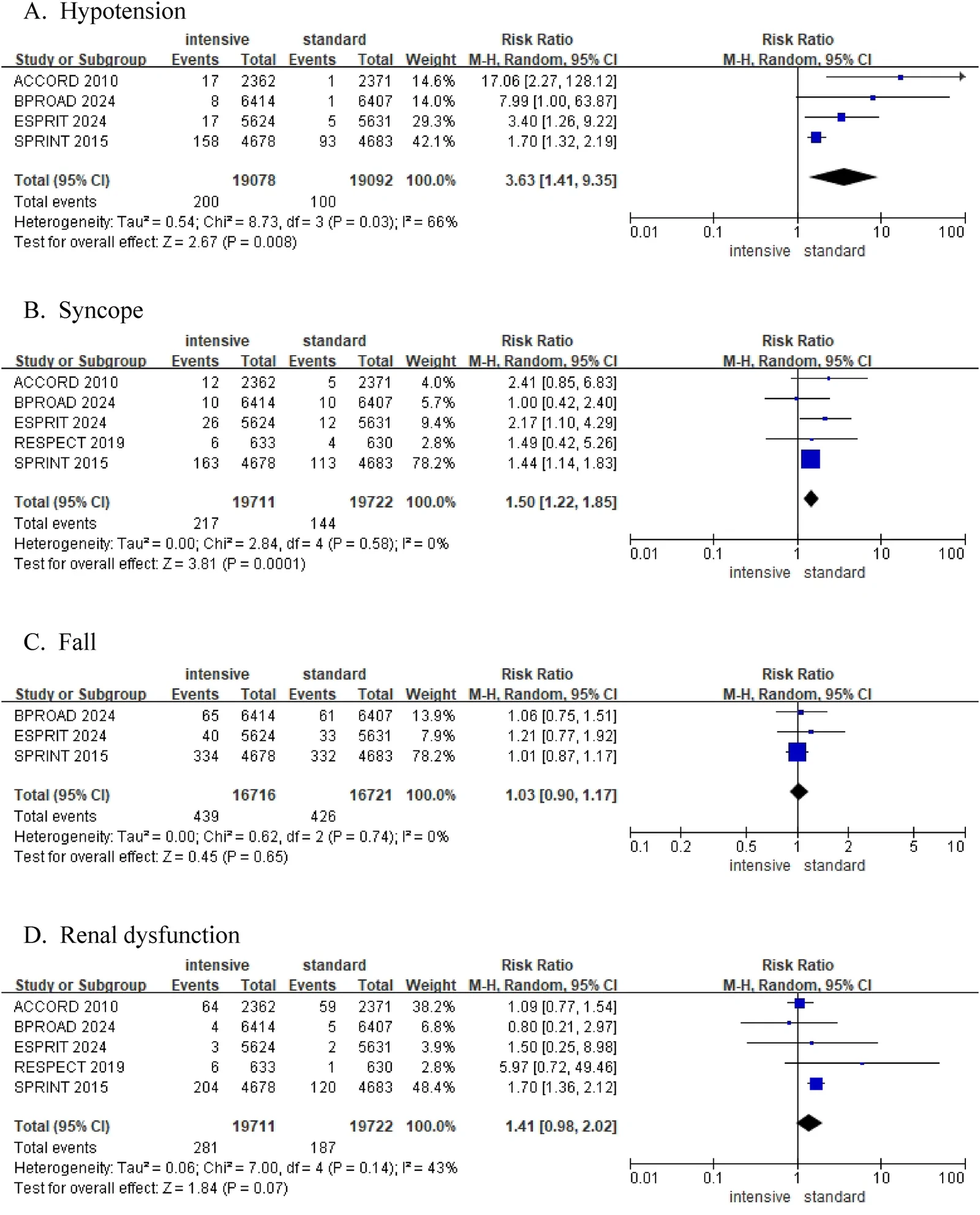
Safety outcomes of intensive blood pressure control. **A**. Hypotension. **B**. Syncope. **C**. Fall. **D**. Renal dysfunction.

### Robustness analyses

Sensitivity analyses confirmed that the pooled estimates for most outcomes were not driven by any single trial (Supplementary Table S7). For all-cause mortality (RR 0.86, 95% CI 0.75–0.99), the leave-one-out RRs ranged from 0.82 to 0.91. When SPRINT was excluded, the estimate became non-significant (RR 0.91, 95% CI 0.79–1.04); similarly, excluding ESPRIT also rendered the estimate non-significant (RR 0.89, 95% CI 0.75–1.05). This suggests that the overall significance of the all-cause mortality finding was partially influenced by these two trials. For cardiovascular mortality (RR 0.73, 95% CI 0.56–0.94), the leave-one-out RRs ranged from 0.65 to 0.78. When SPRINT, BPROAD, or ESPRIT was excluded, the estimate no longer reached statistical significance, indicating that the cardiovascular mortality finding is less robust and should be interpreted with caution.

The results of the TSA are presented in Supplementary Figure S2. For major adverse cardiovascular events, the evidence for a beneficial effect is sufficient and robust against random error. Similarly, both heart failure and stroke showed robust beneficial effects. In contrast, the current evidence for all-cause mortality remains insufficient to draw a definitive conclusion. For cardiovascular mortality, the evidence is inconclusive, and further trials are needed.

### Subgroup analysis in type 2 diabetes patients

Among patients with type 2 diabetes, intensive blood pressure control significantly reduced the risk of major adverse cardiovascular events (RR, 0.82; 95%CI, 0.75–0.90; *P* < 0.001; I^2^ = 6%) and stroke (RR, 0.72; 95%CI, 0.54–0.96; *P* = 0.03; I^2^ = 50%), although it showed no significant benefit for myocardial infarction(RR, 0.87; 95%CI, 0.72–1.04; *P* = 0.14; I^2^ = 0%), all-cause mortality (RR, 0.99; 95%CI, 0.85–1.15; *P* = 0.89; I^2^ = 0%), cardiovascular mortality(RR, 0.88; 95%CI, 0.65–1.20; *P* = 0.42; I^2^ = 37%), and heart failure (RR, 0.83; 95%CI, 0.62–1.12; *P* = 0.22; I^2^ = 25%) (Figure 8).

**Figure 8:**
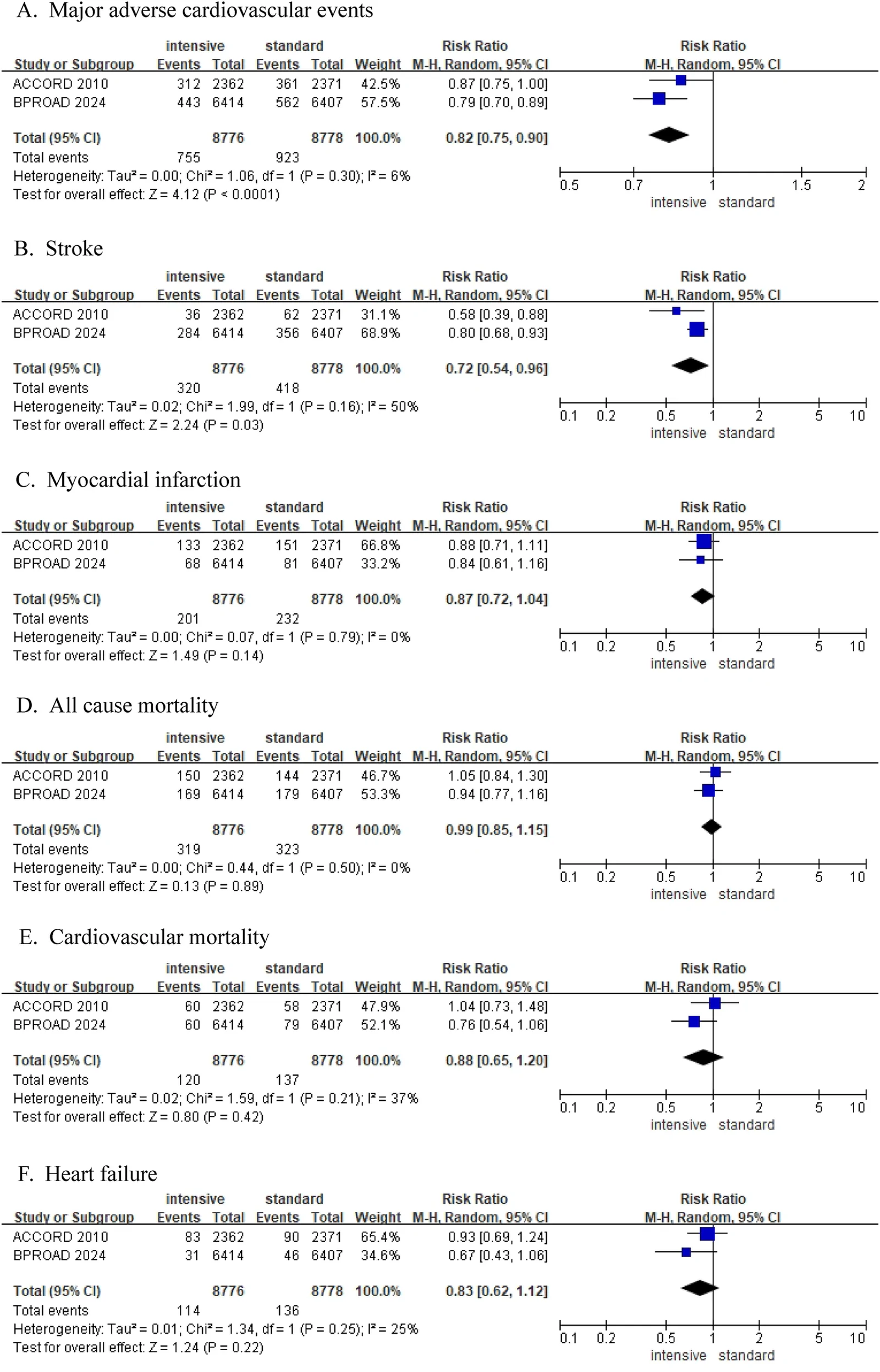
Intensive blood pressure control in patients with type 2 diabetes. **A**. Major adverse cardiovascular events. **B**. Stroke. **C**. Myocardial infarction. **D**. All cause mortality. **E**. Cardiovascular mortality. **F**. Heart failure.

## Discussion

Despite decades of evidence demonstrating that antihypertensive therapy effectively reduces cardiovascular risk (30, 31), the optimal blood pressure treatment target remains a subject of significant debate. This controversy persists even after major guidelines incorporated findings from trials like SPRINT to lower diagnostic and therapeutic thresholds. As far as we know, few meta-analyses have directly compared intensive vs. standard blood pressure control for comprehensive cardiovascular efficacy and safety outcomes. Our analysis included five large-scale, high-quality randomized controlled trials that are pivotal in the field of intensive blood pressure control. The results indicate that intensive blood pressure control to below 120 mmHg significantly reduces the risk of major adverse cardiovascular events compared to a standard target of 140 mmHg. This finding is particularly noteworthy because it suggests that achieving a systolic blood pressure level within the normal range (commonly considered 100–120 mmHg) confers additional cardiovascular benefit. Historically, the treatment targets for hypertensive patients are set below 140 mmHg, which is higher than the above normal range. Our findings suggest that intensive systolic blood pressure lowering may confer cardiovascular benefits in selected high-risk populations. However, several lines of evidence warrant a cautious interpretation of the mortality benefit. First, in the sensitivity analysis, the statistical significance of cardiovascular mortality was partially dependent on the inclusion of specific trials; excluding SPRINT, BPROAD, or ESPRIT rendered the estimate non-significant, suggesting that the finding was not robust to the exclusion of certain studies. Second, TSA further contextualized this uncertainty. For cardiovascular mortality, the current evidence remains inconclusive and may be subject to random error. For all-cause mortality, the evidence for mortality reduction is insufficient to draw a definitive conclusion. In contrast, TSA confirmed that the evidence for major adverse cardiovascular events, stroke, and heart failure was robust. These findings support that while intensive blood pressure lowering confers consistent benefits on composite cardiovascular endpoints and heart failure, its effect on mortality, remains less certain and should be considered hypothesis generating rather than definitive, particularly cardiovascular mortality. This evidence offers a new perspective for refining future hypertension management strategies and guideline recommendations. The inconclusive TSA results for mortality underscore the need for additional large-scale trials with adequate power and longer follow-up to confirm whether intensive blood pressure lowering translates into a survival benefit. Until then, treatment decisions should be individualized, carefully weighing the established cardiovascular benefits against the potential for treatment-related adverse events, and taking into account patient characteristics, comorbidities, and preferences.

Among the included trials, both the ACCORD and BPROAD specifically enrolled patients with type 2 diabetes (21, 29). Our subgroup meta-analysis of these two studies demonstrated that intensive blood pressure control was associated with a significant reduction in major adverse cardiovascular events and stroke. However, no significant benefits were observed for myocardial infarction, heart failure, all-cause mortality, or cardiovascular mortality. The neutral findings for mortality outcomes warrant particular attention, as they contrast with the significant mortality benefits observed in the overall population (which included non-diabetic patients). Several factors may explain the attenuated treatment effect in patients with diabetes. First, the actual event rate in the standard group of ACCORD was lower than anticipated during the trial design, further limiting its power to detect a benefit. Second, the ACCORD trial employed a 2 × 2 factorial design that included both intensive and standard glycemic control arms. A pre-specified subgroup analysis demonstrated that the cardiovascular benefit of intensive blood pressure lowering was significant only among patients assigned to standard glycemic control, but not among those receiving intensive glycemic management, suggesting that intensive glucose lowering may have attenuated or confounded the additional benefit of further blood pressure reduction (32). Third, the pathophysiology of cardiovascular disease in patients with diabetes is more complex, involving not only hemodynamic factors but also metabolic dysregulation, inflammation, and accelerated atherosclerosis, which may diminish the incremental benefit of more aggressive blood pressure lowering. Fourth, the diabetes subgroup analysis was based on only two trials and was not powered for subgroup comparisons, as reflected in the wide confidence intervals for several outcomes. Despite the reduction in major adverse cardiovascular events and stroke, the absence of a clear mortality benefit in diabetic patients underscores the need for caution in extrapolating findings from non-diabetic populations to patients with diabetes. Our findings suggest that while intensive blood pressure lowering may reduce certain cardiovascular endpoints in diabetic patients, the overall benefit-risk balance remains uncertain. Future dedicated trials with adequate power and longer follow-up are needed to define the optimal blood pressure target in this population.

Elevated blood pressure is a particularly strong risk factor for stroke in Asian populations. It is reported that the association between systolic blood pressure and stroke is more pronounced in Asian than in Australasian populations (33). After age standardization, a 10-mmHg reduction in systolic blood pressure was associated with a 41% reduction in stroke risk in Asia, compared with 30% in Australasia. Given that stroke is the leading cause of cardiovascular mortality in China (34), its prevention is a paramount goal in hypertension management. Our analysis contributes to the understanding of stroke prevention through intensive blood pressure control. While the SPRINT trial excluded individuals with prior stroke, the RESPECT study specifically enrolled this population. Results from a secondary analysis of the SPRINT study indicate that, compared with standard blood pressure-lowering therapy, intensive blood pressure-lowering therapy was associated with increased rather than decreased cerebral perfusion; this effect was particularly pronounced among participants with a history of cardiovascular disease (35). The INFINITY study, which excluded patients with a previous stroke, examined the effects of two different 24-hour mean systolic blood pressure targets (<130 mmHg vs. <145 mmHg) on mobility, white matter disease progression, and cognitive function over three years (36). The study found that intensive outpatient blood pressure control in elderly patients with hypertension, while not improving their functional capacity, was associated with a reduced accumulation of subcortical white matter lesions. A reduction in the accumulation of white matter disease over periods longer than three years may help conserve function. In patients with a history of stroke, the RESPECT study found that compared to a standard target of below 140 mmHg, intensive systolic blood pressure lowering to below120 mmHg did not significantly reduce the risk of overall stroke recurrence but was associated with a lower risk of intracranial hemorrhage. Similarly, the SPS3 trial, a key study in this field, supported a systolic blood pressure target of below 130 mmHg for preventing recurrent stroke, particularly in patients with recent lacunar infarcts (13). The ESPRIT study, which included approximately 25% of participants with a history of stroke, provided further insights through a prespecified secondary analysis. This analysis indicated that compared with systolic blood pressure below 140 mmHg, maintaining systolic blood pressure below 120 mmHg reduced the risk of hemorrhagic stroke by 50% without a corresponding increase in the risk of ischemic stroke (37). These findings from individual trials suggest that the benefit of intensive blood pressure control for preventing overall stroke recurrence may be less pronounced than for primary prevention, though a reduction in hemorrhagic stroke risk appears evident.

Hypertension is a common chronic disease among the elderly and is a major risk factor for cardiovascular and cerebrovascular diseases, chronic kidney diseases and mortality among the elderly (38, 39). It is estimated that the prevalence of hypertension exceeds 60% among people aged 60 and older (40). Elderly patients often present with multiple health issues, including chronic diseases or functional impairments. It is worth noting that the elderly constitute a distinct population group, and their blood pressure management methods may differ from those of the general population. Results from the SPRINT study indicated that among ambulatory adults aged 75 and older, the incidence of fatal and non-fatal major cardiovascular events and death from any cause was significantly reduced when the systolic blood pressure target was below 120 mmHg compared with that below 140 mmHg (41). But our meta-analysis results demonstrate that intensive blood pressure control significantly increases the risk of adverse events such as hypotension, syncope, and renal dysfunction. This finding aligns with previous studies which have also reported that intensive antihypertensive treatment is associated with an elevated risk of these adverse events (17, 42, 43). Arterial blood pressure serves as the driving force for organ perfusion; excessively low pressure can lead to systemic hypoperfusion. While hypertension is detrimental, hypotension can likewise harm various organs. For instance, hypotension may increase the risk of dementia and Parkinson's disease in older adults by reducing cerebral perfusion pressure (44, 45). Moreover, excessively low blood pressure has been associated with an increased risk of all-cause mortality in old women (46). This risk pattern is exemplified by a study of the oldest-old in China, which revealed a U-shaped association between systolic blood pressure and three-year all-cause mortality. Specifically, higher systolic blood pressure was linked to cardiovascular death, whereas lower systolic blood pressure correlated with non-cardiovascular mortality (47). These findings demonstrate that the “lower-is-better” premise cannot be universally applied, particularly among the oldest-old population. The patient subgroups most likely to derive net clinical benefit from intensive blood pressure control include middle-aged, relatively healthy adults with hypertension, as well as individuals at high cardiovascular risk. Therefore, implementing intensive blood pressure control, a comprehensive individual assessment is essential to ensure that the potential benefits outweigh the risks. Rigorous participant screening, monitoring for orthostatic hypotension and syncope, and periodic evaluation of treatment tolerance constitute essential prerequisites for the safe clinical implementation of intensive blood pressure control strategies.

Cardiovascular medicine has entered the era of cardiometabolic medicine. The current focus has shifted from merely preventing acute events like heart attacks to addressing the four major, interrelated epidemics that define disease risk: obesity, diabetes, chronic kidney disease, and hypertension (48). If the past half-century can be termed the “era of arteries,” the future may be the “era of metabolism”. This conceptual shift is reflected in our evolving understanding of hypertension: once viewed primarily as a pathway to heart failure through vascular damage, it is now recognized as being intricately intertwined with diabetes and obesity within a shared metabolic ecosystem. Recent studies further highlight a broader transition in the field—from acute events to chronic comorbidities, from hemodynamics to metabolism, and from short-term survival to long-term quality of life (48, 49). Concurrently, epidemiological trends underscore this evolution. While deaths from cardiovascular disease have declined significantly, non-cardiovascular deaths are rising. Although survival rates for patients with heart failure have improved significantly, an increasing number of these patients suffer from obesity, diabetes, and chronic kidney disease. Data from an analysis of over 80,000 participants in the US National Health and Nutrition Examination Survey illustrate this shift clearly: among heart failure patients, the prevalence of obesity rose from 32.5% in 1988 to 60.4% in 2023, impaired glucose homeostasis increased from 48.6% to 69.2%, diabetes from 21.2% to 36.2%, and chronic kidney disease from 38.6% to 52.3%. In contrast, the prevalence of hypertension decreased from 80.7% to 49.1%, hypercholesterolemia from 71.5% to 22.6%, and a history of myocardial infarction from 59.3% to 42.1% (49). Consequently, heart failure is transitioning from an ischemic-dominated to a metabolism-dominated entity, with its core pathology evolving from arterial occlusion to systemic metabolic dysfunction—essentially becoming a metabolic disease. Further supporting this paradigm, Lee et al. analyzed data from 21,822 adults with hypertension between 1999 and 2023 (50). Their national portrait of cardiometabolic multimorbidity revealed that hypertension is now deeply embedded within the triad of diabetes, hyperlipidemia, and obesity. Specifically, the proportion of hypertensive patients with diabetes increased from 17.2% in 1999 to 27.8% in 2023. Notably, 75% of these patients also had hyperlipidemia, and 20% had both diabetes and hyperlipidemia. Moreover, over the past decade, the proportion achieving target ranges for all three key metrics—blood pressure, blood glucose, and blood lipids—has remained consistently below 30%, indicating a plateau in comprehensive metabolic control. These findings collectively underscore a critical realization: hypertension is no longer merely an issue of elevated blood pressure but an integral component of metabolic syndrome that demands holistic management. This evidence necessitates a fundamental shift in clinical practice—from disease-specific strategies to a patient-centered, integrated care model that recognizes the indivisibility of cardiovascular, renal, and metabolic health.

## Limitation

Our study has several limitations. First, although all included trials employed randomized controlled designs, the nature of the intervention (which required patients to actively participate in blood pressure measurements) made it impossible to blind participants and researchers, thereby introducing potential performance bias. Second, while the systolic blood pressure targets were uniform across trials, notable heterogeneity was observed in the specific antihypertensive regimens. Different drug classes may exert varying effects on specific cardiovascular outcomes, which may confound the observed treatment effect. Third, non-English trials were excluded, and we did not systematically include trial registries, grey literature, or unpublished studies, this may affect the comprehensiveness of our pooled results. Fourth, due to the limited number of included studies, obvious clinical heterogeneity among enrolled participants, and the fact that several pooled outcomes were dependent on a small subset of trials, further research is needed to determine whether our findings can be generalized to all patients with hypertension.

## Conclusion

This meta-analysis demonstrates that intensive systolic blood pressure control (target SBP <120 mmHg) may significantly reduce cardiovascular and mortality risks compared to standard blood pressure control (target SBP <140 mmHg). However, these benefits must be weighed against the increased risks of adverse events, including hypotension and syncope. Importantly, our findings do not support a universal SBP target of <120 mmHg for all patients; rather, treatment decisions should be individualized based on patient characteristics, comorbidities, and tolerability. Given hypertensive patients frequently present with multiple comorbidities, the efficacy and safety of intensive blood pressure control may vary across patients with distinct concomitant diseases, it is necessary to conduct rigorously designed clinical trials to verify these findings.

## Data Availability

The original contributions presented in the study are included in the article/Supplementary Material, further inquiries can be directed to the corresponding author/s.
