# Supplementary material for: Efficacy and safety of intensive vs. standard blood pressure control: a systematic review and meta-analysis
Source: Front Cardiovasc Med. 2026 Sep 23;13:1929546. doi: 10.3389/fcvm.2026.1929546 (PMC13642591; doi:10.3389/fcvm.2026.1929546)
Supplement: Supplementary file 1 [file Supplementaryfile1.doc]

**Supplementary material**

Table S1 Literature retrieval strategy of PubMed database	2

Table S2 Literature retrieval strategy of Embase database	6

Table S3 Literature retrieval strategy of Cochrane database	7

Table S4 Outcome-specific trial inclusions and exclusions	8

Table S5 GRADE summary of findings	9

Table S6 Absolute effect estimates and numbers needed to treat/harm	11

Table S7 Sensitivity analysis	12

Figure S1 Risk of bias summary/graph of included literature-the updated Cochrane RoB 2.0 tool	13

Figure S2 Trial sequential analysis	14

# Table S1 Literature retrieval strategy of PubMed database

| Step | Search Query | Hits |
| --- | --- | --- |
| #1 | "hypertension"[MeSH Terms] OR "high blood pressure"[Title/Abstract] OR "blood pressure high"[Title/Abstract] OR "essential hypertension"[Title/Abstract] OR "systolic hypertension"[Title/Abstract] OR "systolic blood pressure"[Title/Abstract] OR "diastolic blood pressure"[Title/Abstract] OR "diastolic hypertension"[Title/Abstract] | 406,138 |
| #2 | "antihypertensive*"[Title/Abstract] OR "Antihypertensive"[Title/Abstract] OR "hypertensive treatment"[Title/Abstract] OR "intensive antihypertensive"[Title/Abstract] OR "intensive blood pressure control"[Title/Abstract] OR "intensive blood pressure control"[Title/Abstract] OR "standard blood pressure control"[Title/Abstract] OR "standard blood pressure control"[Title/Abstract] OR "blood pressure control"[Title/Abstract] OR "blood pressure control"[Title/Abstract] OR "Standard-treatment"[Title/Abstract] OR "intensive therapy"[Title/Abstract] OR "120mmHg"[Title/Abstract] OR "140 mmhg"[Title/Abstract] | 118,353 |
| #3 | "cardiovascular events"[Title/Abstract] OR "major cardiovascular events"[Title/Abstract] OR "cardiovascular"[Title/Abstract] OR "myocardial infarction"[Title/Abstract] OR "infarction"[Title/Abstract] OR "stroke"[Title/Abstract] OR "death"[Title/Abstract] OR "mortality"[Title/Abstract] OR "heart failure"[Title/Abstract] OR "hospitalization"[Title/Abstract] OR "hypotension"[Title/Abstract] OR "adverse events"[Title/Abstract] | 3,181,946 |
| #4 | ("hypertension"[MeSH Terms] OR "high blood pressure"[Title/Abstract] OR "blood pressure high"[Title/Abstract] OR "essential hypertension"[Title/Abstract] OR "systolic hypertension"[Title/Abstract] OR "systolic blood pressure"[Title/Abstract] OR "diastolic blood pressure"[Title/Abstract] OR "diastolic hypertension"[Title/Abstract]) AND ("antihypertensive*"[Title/Abstract] OR "Antihypertensive"[Title/Abstract] OR "hypertensive treatment"[Title/Abstract] OR "intensive antihypertensive"[Title/Abstract] OR "intensive blood pressure control"[Title/Abstract] OR "intensive blood pressure control"[Title/Abstract] OR "standard blood pressure control"[Title/Abstract] OR "standard blood pressure control"[Title/Abstract] OR "blood pressure control"[Title/Abstract] OR "blood pressure control"[Title/Abstract] OR "Standard-treatment"[Title/Abstract] OR "intensive therapy"[Title/Abstract] OR "120mmHg"[Title/Abstract] OR "140 mmhg"[Title/Abstract]) | 48,291 |
| #5 | ("hypertension"[MeSH Terms] OR "high blood pressure"[Title/Abstract] OR "blood pressure high"[Title/Abstract] OR "essential hypertension"[Title/Abstract] OR "systolic hypertension"[Title/Abstract] OR "systolic blood pressure"[Title/Abstract] OR "diastolic blood pressure"[Title/Abstract] OR "diastolic hypertension"[Title/Abstract]) AND ("antihypertensive*"[Title/Abstract] OR "Antihypertensive"[Title/Abstract] OR "hypertensive treatment"[Title/Abstract] OR "intensive antihypertensive"[Title/Abstract] OR "intensive blood pressure control"[Title/Abstract] OR "intensive blood pressure control"[Title/Abstract] OR "standard blood pressure control"[Title/Abstract] OR "standard blood pressure control"[Title/Abstract] OR "blood pressure control"[Title/Abstract] OR "blood pressure control"[Title/Abstract] OR "Standard-treatment"[Title/Abstract] OR "intensive therapy"[Title/Abstract] OR "120mmHg"[Title/Abstract] OR "140 mmhg"[Title/Abstract]) AND ("cardiovascular events"[Title/Abstract] OR "major cardiovascular events"[Title/Abstract] OR "cardiovascular"[Title/Abstract] OR "myocardial infarction"[Title/Abstract] OR "infarction"[Title/Abstract] OR "stroke"[Title/Abstract] OR "death"[Title/Abstract] OR "mortality"[Title/Abstract] OR "heart failure"[Title/Abstract] OR "hospitalization"[Title/Abstract] OR "hypotension"[Title/Abstract] OR "adverse events"[Title/Abstract]) | 20,105 |
| #6 | "randomized controlled trial"[Publication Type] | 632,847 |
| #7 | "randomized controlled trial"[Publication Type] AND (("hypertension"[MeSH Terms] OR "high blood pressure"[Title/Abstract] OR "blood pressure high"[Title/Abstract] OR "essential hypertension"[Title/Abstract] OR "systolic hypertension"[Title/Abstract] OR "systolic blood pressure"[Title/Abstract] OR "diastolic blood pressure"[Title/Abstract] OR "diastolic hypertension"[Title/Abstract]) AND ("antihypertensive*"[Title/Abstract] OR "Antihypertensive"[Title/Abstract] OR "hypertensive treatment"[Title/Abstract] OR "intensive antihypertensive"[Title/Abstract] OR "intensive blood pressure control"[Title/Abstract] OR "intensive blood pressure control"[Title/Abstract] OR "standard blood pressure control"[Title/Abstract] OR "standard blood pressure control"[Title/Abstract] OR "blood pressure control"[Title/Abstract] OR "blood pressure control"[Title/Abstract] OR "Standard-treatment"[Title/Abstract] OR "intensive therapy"[Title/Abstract] OR "120mmHg"[Title/Abstract] OR "140 mmhg"[Title/Abstract]) AND ("cardiovascular events"[Title/Abstract] OR "major cardiovascular events"[Title/Abstract] OR "cardiovascular"[Title/Abstract] OR "myocardial infarction"[Title/Abstract] OR "infarction"[Title/Abstract] OR "stroke"[Title/Abstract] OR "death"[Title/Abstract] OR "mortality"[Title/Abstract] OR "heart failure"[Title/Abstract] OR "hospitalization"[Title/Abstract] OR "hypotension"[Title/Abstract] OR "adverse events"[Title/Abstract])) | 2,371 |
| #8 | "English"[Language] | 33,511,658 |
| #9 | "English"[Language] AND ("randomized controlled trial"[Publication Type] AND (("hypertension"[MeSH Terms] OR "high blood pressure"[Title/Abstract] OR "blood pressure high"[Title/Abstract] OR "essential hypertension"[Title/Abstract] OR "systolic hypertension"[Title/Abstract] OR "systolic blood pressure"[Title/Abstract] OR "diastolic blood pressure"[Title/Abstract] OR "diastolic hypertension"[Title/Abstract]) AND ("antihypertensive*"[Title/Abstract] OR "Antihypertensive"[Title/Abstract] OR "hypertensive treatment"[Title/Abstract] OR "intensive antihypertensive"[Title/Abstract] OR "intensive blood pressure control"[Title/Abstract] OR "intensive blood pressure control"[Title/Abstract] OR "standard blood pressure control"[Title/Abstract] OR "standard blood pressure control"[Title/Abstract] OR "blood pressure control"[Title/Abstract] OR "blood pressure control"[Title/Abstract] OR "Standard-treatment"[Title/Abstract] OR "intensive therapy"[Title/Abstract] OR "120mmHg"[Title/Abstract] OR "140 mmhg"[Title/Abstract]) AND ("cardiovascular events"[Title/Abstract] OR "major cardiovascular events"[Title/Abstract] OR "cardiovascular"[Title/Abstract] OR "myocardial infarction"[Title/Abstract] OR "infarction"[Title/Abstract] OR "stroke"[Title/Abstract] OR "death"[Title/Abstract] OR "mortality"[Title/Abstract] OR "heart failure"[Title/Abstract] OR "hospitalization"[Title/Abstract] OR "hypotension"[Title/Abstract] OR "adverse events"[Title/Abstract]))) | 2,282 |

# Table S2 Literature retrieval strategy of Embase database

| Step | Search Query | Hits |
| --- | --- | --- |
| #1 | 'hypertension'/exp OR hypertension OR 'high blood pressure':ab,ti OR 'blood pressure, high':ab,ti OR 'essential hypertension':ab,ti OR 'systolic hypertension':ab,ti OR 'systolic blood pressure':ab,ti OR 'diastolic blood pressure':ab,ti OR 'diastolic hypertension':ab,ti | 1,805,687 |
| #2 | 'antihypertensive agent'/exp OR 'antihypertensive agent' OR antihypertensive:ab,ti OR 'hypertension treatment':ab,ti OR 'intensive antihypertensive':ab,ti OR 'intensive blood-pressure control':ab,ti OR 'intensive blood pressure control':ab,ti OR 'standard blood-pressure control':ab,ti OR 'standard blood pressure control':ab,ti OR 'blood-pressure control':ab,ti OR 'blood pressure control':ab,ti OR 'standard treatment':ab,ti OR 'intensive therapy':ab,ti OR 120mmhg:ab,ti OR '140 mmHg':ab,ti | 1,352,425 |
| #3 | 'cardiovascular events' OR (('cardiovascular'/exp OR 'cardiovascular') AND 'events') OR 'major cardiovascular events':ab,ti OR cardiovascular:ab,ti OR 'myocardial infarction':ab,ti OR infarction:ab,ti OR stroke:ab,ti OR death:ab,ti OR mortality:ab,ti OR 'heart failure':ab,ti OR hospitalization:ab,ti OR 'adverse events':ab,ti | 5,291,636 |
| #4 | english:la | 43,382,392 |
| #5 | 'randomized controlled trial':ab,ti | 233,265 |
| #6 | #1 AND #2 AND #3 AND #4 AND #5 | 1,155 |

# Table S3 Literature retrieval strategy of Cochrane database

| Step | Search Query | Hits |
| --- | --- | --- |
| #1 | `("hypertension"):ti,ab,kw OR ("High blood pressure"):ti,ab,kw OR ("Blood pressure, high"):ti,ab,kw OR ("Essential hypertension"):ti,ab,kw OR ("Systolic hypertension"):ti,ab,kw | 104,331 |
| #2 | `("Systolic blood pressure"):ti,ab,kw OR ("Diastolic blood pressure"):ti,ab,kw OR ("Diastolic hypertension"):ti,ab,kw | 53,255 |
| #3 | #1 OR #2 | 127,772 |
| #4 | `("cardiovascular events"):ti,ab,kw OR ("major cardiovascular events"):ti,ab,kw OR ("cardiovascular"):ti,ab,kw OR ("myocardial infarction"):ti,ab,kw OR ("infarction"):ti,ab,kw | 132,016 |
| #5 | `("stroke"):ti,ab,kw OR ("death"):ti,ab,kw OR ("mortality"):ti,ab,kw OR ("heart failure"):ti,ab,kw OR ("hospitalization"):ti,ab,kw | 294,352 |
| #6 | `("hypotension"):ti,ab,kw OR ("adverse events"):ti,ab,kw | 184,695 |
| #7 | #4 OR #5 OR #6 | 497,946 |
| #8 | `("randomized controlled trial"):ti,ab,kw | 695,349 |
| #9 | English:la | 2,133,454 |
| #10 | `("Intensive antihypertensive"):ti,ab,kw OR ("Intensive blood-pressure control"):ti,ab,kw OR ("Intensive blood pressure control"):ti,ab,kw OR ("Intensive therapy"):ti,ab,kw OR ("120mmHg"):ti,ab,kw | 32,174 |
| #11 | #10 AND #3 AND #7 AND #8 AND #9 | 1,574 |

# Table S4 Outcome-specific trial inclusions and exclusions

| Outcome | Included Trials | Excluded Trials | Reason for Exclusion |
| --- | --- | --- | --- |
| MACE | SPRINT, ACCORD, ESPRIT, BPROAD | RESPECT | Heart failure and cardiovascular mortality data not reported |
| Myocardial infarction | SPRINT, ACCORD, ESPRIT, BPROAD, RESPECT | — | — |
| Stroke | SPRINT, ACCORD, ESPRIT, BPROAD, RESPECT | — | — |
| Heart failure | SPRINT, ACCORD, ESPRIT, BPROAD | RESPECT | Heart failure data not reported |
| All-cause mortality | SPRINT, ACCORD, ESPRIT, BPROAD, RESPECT | — | — |
| Cardiovascular mortality | SPRINT, ACCORD, ESPRIT, BPROAD | RESPECT | Cardiovascular mortality data not separately reported |
| Hypotension | SPRINT, ACCORD, ESPRIT, BPROAD | RESPECT | Hypotension data not reported |
| Syncope | SPRINT, ACCORD, ESPRIT, BPROAD, RESPECT | — | — |
| Falls | SPRINT, ESPRIT, BPROAD | RESPECT, ACCORD | Falls data not reported |
| Renal dysfunction | SPRINT, ACCORD, ESPRIT, BPROAD, RESPECT | — | — |

Note: MACE, major cardiovascular events.

# Table S5 GRADE summary of findings

| **Intensive vs Standard Blood Pressure Control** | | | | | |
| --- | --- | --- | --- | --- | --- |
| **Patient or population:** hypertension  **Setting:** randomized controlled trials  **Intervention:** intensive  **Comparison:** standard | | | | | |
| **Outcomes** | **№ of participants (studies) Follow-up** | **Certainty of the evidence (GRADE)** | **Relative effect (95% CI)** | **Anticipated absolute effects** | |
|  |  |  |  | **Risk with standard** | **Risk difference with intensive** |
| MACE (MACE) assessed with: a composite of major adverse cardiovascular events, including fatal or non-fatal heart failure, myocardial infarction, stroke, and cardiovascular mortality.  follow-up: mean 3.8 years | 38170 (4 RCTs) | ⨁⨁⨁⨁ High^a^ | **RR 0.80** (0.75 to 0.86) | 97 per 1,000 | **19 fewer per 1,000** (from 24 fewer to 14 fewer) |
| heart failure (heart failure) assessed with: Heart failure events that require hospitalization or emergency treatment follow-up: mean 3.8 years | 38170 (4 RCTs) | ⨁⨁⨁⨁ High^a^ | **RR 0.74** (0.62 to 0.90) | 16 per 1,000 | **4 fewer per 1,000** (from 6 fewer to 2 fewer) |
| stroke (stroke) assessed with: Ischemic or hemorrhagic stroke follow-up: mean 3.9 years | 39433 (5 RCTs) | ⨁⨁⨁⨁ High^a^ | **RR 0.81** (0.73 to 0.90) | 43 per 1,000 | **8 fewer per 1,000** (from 12 fewer to 4 fewer) |
| myocardial infarction (myocardial infarction) assessed with: Myocardial infarction that conforms to the general definition follow-up: mean 3.9 years | 39433 (5 RCTs) | ⨁⨁⨁⨁ High^a^ | **RR 0.87** (0.76 to 1.00) | 22 per 1,000 | **3 fewer per 1,000** (from 5 fewer to 0 fewer) |
| All cause mortality (All cause mortality) assessed with: all cause mortality follow-up: mean 3.9 years | 39433 (5 RCTs) | ⨁⨁⨁⨁ High^a^ | **RR 0.86** (0.75 to 0.99) | 39 per 1,000 | **5 fewer per 1,000** (from 10 fewer to 0 fewer) |
| Cardiovascular mortality (Cardiovascular mortality) assessed with: Death caused by cardiovascular issues follow-up: mean 3.8 years | 38170 (4 RCTs) | ⨁⨁⨁◯ Moderate^a,b^ | **RR 0.73** (0.56 to 0.94) | 16 per 1,000 | **4 fewer per 1,000** (from 7 fewer to 1 fewer) |
| hypotension (hypotension) assessed with: Treatment-related hypotensive events (requiring medical intervention) follow-up: mean 3.8 years | 38170 (4 RCTs) | ⨁⨁⨁◯ Moderate^a,b^ | **RR 3.63** (1.41 to 9.35) | 5 per 1,000 | **14 more per 1,000** (from 2 more to 44 more) |
| syncope (syncope) assessed with: Syncope incident follow-up: mean 3.9 years | 39433 (5 RCTs) | ⨁⨁⨁⨁ High^a^ | **RR 1.50** (1.22 to 1.85) | 7 per 1,000 | **4 more per 1,000** (from 2 more to 6 more) |
| fall (fall) assessed with: Falling incident (requiring medical attention) follow-up: mean 3.4 years | 33437 (3 RCTs) | ⨁⨁⨁⨁ High^a^ | **RR 1.03** (0.90 to 1.17) | 25 per 1,000 | **1 more per 1,000** (from 3 fewer to 4 more) |
| renal dysfunction (renal dysfunction) assessed with: According to the definitions of renal dysfunction events as specified in each trial protocol follow-up: mean 3.9 years | 39433 (5 RCTs) | ⨁⨁⨁⨁ High^a^ | **RR 1.41** (0.98 to 2.02) | 9 per 1,000 | **4 more per 1,000** (from 0 fewer to 10 more) |
| ***The risk in the intervention group** (and its 95% confidence interval) is based on the assumed risk in the comparison group and the **relative effect** of the intervention (and its 95% CI).  **CI:** confidence interval; **RR:** risk ratio | | | | | |
| **GRADE Working Group grades of evidence** **High certainty:** we are very confident that the true effect lies close to that of the estimate of the effect. **Moderate certainty:** we are moderately confident in the effect estimate: the true effect is likely to be close to the estimate of the effect, but there is a possibility that it is substantially different. **Low certainty:** our confidence in the effect estimate is limited: the true effect may be substantially different from the estimate of the effect. **Very low certainty:** we have very little confidence in the effect estimate: the true effect is likely to be substantially different from the estimate of effect. | | | | | |

#### **Explanations**

a. Participants were not blinded to their group allocation in any of the included studies, but we think this will not affect the results.

b. I2 is more than 50%

# Table S6 Absolute effect estimates and numbers needed to treat/harm

| Outcome | Mean Follow-up (years) | Absolute Risk Difference (ARD) per 1,000 (95% CI) | NNT or NNH (95% CI)* |
| --- | --- | --- | --- |
| **Efficacy outcomes** |  |  |  |
| MACE | 3.8 | 19 fewer (14 to 24 fewer) | NNT = 53 (41 to 77) |
| Stroke | 3.9 | 8 fewer (4 to 12 fewer) | NNT = 125 (83 to 250) |
| Heart failure | 3.8 | 4 fewer (2 to 6 fewer) | NNT = 250 (167 to 500) |
| Myocardial infarction | 3.9 | 3 fewer (0 to 5 fewer) | NNT = 333 (200 to ∞)† |
| All-cause mortality | 3.9 | 5 fewer (0 to 10 fewer) | NNT = 200 (100 to ∞)† |
| Cardiovascular mortality | 3.8 | 4 fewer (1 to 7 fewer) | NNT = 250 (143 to 1,000) |
| **Safety outcomes** |  |  |  |
| Hypotension | 3.8 | 14 more (2 to 44 more) | NNH = 71 (50 to 125) |
| Syncope | 3.9 | 4 more (2 to 6 more) | NNH = 250 (167 to 500) |
| Renal dysfunction | 3.9 | 4 more (0 to 10 more) | NNH = 250 (100 to ∞)† |
| Falls | 3.4 | 1 more (3 fewer to 4 more) | Not calculated‡ |

*NNT/NNH were calculated as the inverse of the absolute risk difference; values are rounded up to the nearest whole number. 95% CIs for NNT/NNH were derived from the confidence limits of the ARD.
†Not statistically significant; NNT/NNH estimates should be interpreted with caution.
‡Not calculated as the relative effect was not statistically significant with a confidence interval crossing unity.

# Table S7 Sensitivity analysis

| Study omitted | Major adverse cardiovascular events | Heart failure | Stroke | Myocardial infarction | All cause mortality | Cardiovascular mortality |
| --- | --- | --- | --- | --- | --- | --- |
| ACCORD 2010 | 0.78[0.73,0.84] | 0.67[0.55,0.82] | 0.83[0.75,0.92] | 0.86[0.73,1.02] | 0.82[0.73,0.92] | 0.65[0.53,0.79] |
| SPRINT 2015 | 0.82[0.76,0.88] | 0.80[0.66,0.98] | 0.80[0.71,0.90] | 0.88[0.76,1.03] | 0.91[0.79,1.04] | 0.78[0.58,1.05] |
| RESPECT 2019 | NA | NA | 0.81[0.72,0.91] | 0.87[0.76,0.99] | 0.87[0.74,1.01] | NA |
| BPROAD 2024 | 0.81[0.74,0.88] | 0.75[0.60,0.96] | 0.81[0.69,0.94] | 0.88[0.76,1.02] | 0.84[0.71,0.99] | 0.71[0.49,1.03] |
| ESPRIT 2024 | 0.80[0.73,0.87] | 0.74[0.57,0.97] | 0.78[0.69,0.88] | 0.86[0.74,1.00] | 0.89[0.75,1.05] | 0.77[0.56,1.07] |

# **Figure S1 Risk of bias summary/graph of included literature-the updated Cochrane RoB 2.0 tool**

# **Figure S2 Trial sequential analysis**

A. Trial sequential analysis plot for MACE

B. Trial sequential analysis plot for heart failure

C. Trial sequential analysis plot for stroke

D. Trial sequential analysis plot for myocardial infarction

E. Trial sequential analysis plot for all cause mortality

F. Trial sequential analysis plot for cardiovascular mortality
